# Coexistence of early gastric cancer and benign submucosal lesions mimic invasive cancer: a retrospective multicenter experience

**DOI:** 10.1186/s12876-023-03044-3

**Published:** 2023-11-23

**Authors:** Huawei Yang, Zhen Li, Zhi Wei, Guodong Li, Yi Li, Shanbin Wu, Rui Ji

**Affiliations:** 1 Department of Gastroenterology, Qilu Hospital of Shandong University, No. 107, Wenhuaxi Road, Jinan, 250012 China; 2 Shandong Second Provincial General Hospital, Jinan, 250022 China; 3 The First Affiliated Hospital of Shandong First Medical University, Jinan, 250014 China

**Keywords:** Early gastric cancer, Submucosal lesions, Endoscopic ultrasonography, Gastritis cystica profunda, Infiltration depth

## Abstract

**Objective:**

To present a study to identify the characteristics of coexisting early gastric cancer (EGC) and benign submucosal lesions, with the aim of reducing the adverse consequences of overdiagnosis and overtreatment.

**Methods:**

In this retrospective study, we searched the endoscopic databases of three tertiary centers. We screened of patients suspected of early gastric cancer submucosal infiltration by conventional endoscopy and ultimately selected for endoscopic submucosal dissection treatment after endoscopic ultrasonography and magnifying endoscopy with narrow-band imaging examination. Patients with coexisting EGC and benign submucosal lesions in histological sections were included. Clinical data and endoscopic images were reviewed. To evaluate the precision of endoscopists’ diagnoses for this type of lesion, eight endoscopists with different experiences were recruited to judge the infiltration depth of these lesions and analyze the accuracy rate.

**Results:**

We screened 520 patients and retrospectively identified 18 EGC patients with an invasive cancer-like morphology. The most common lesion site was the cardia (12/18, 66.67%). The coexisting submucosal lesions could be divided into solid (5/18, 27.78%) and cystic (13/18, 72.22%). The most common type of submucosal lesion was gastritis cystica profunda (12/18, 66.67%), whereas leiomyoma was the predominant submucosal solid lesion (3/18, 16.67%). Ten (55.56%) patients < underwent endoscopic ultrasonography; submucosal lesions were definitively diagnosed in 6 patients (60.00%). The accuracy of judgement of the infiltration depth was significantly lower in cases of coexistence of EGC with benign submucosal lesions (EGC-SML) than in EGC (38.50% versus 65.60%, *P* = 0.0167). The rate of over-diagnosis was significantly higher within the EGC-SML group compared to the EGC group (59.17% versus 10.83%, *P* < 0.0001).

**Conclusions:**

We should be aware of the coexistence of EGC and benign submucosal lesions, the most common of which is early cardiac-differentiated cancer with gastritis cystica profunda.

## Introduction

Endoscopic submucosal dissection (ESD) has been established as a first-line treatment modality for selected early gastric cancer (EGC). Whether EGC can be treated endoscopically depends mainly on the risk of lymph node metastasis, which correlates with the invasion depth of the tumor [1]. Therefore, accurate prediction of the tumor invasion depth is of great importance in planning an appropriate treatment strategy and promising curative resection [2]. Computed tomography (CT), magnetic resonance imaging (MRI), and positron emission tomography are mainly used to evaluate advanced gastric cancer, but these methods are not accurate in predicting the infiltration depth of EGC [3]. Currently, judgement of the infiltration depth of mucosal neoplastic lesions relies on white-light endoscopy (WLE), magnifying endoscopy with narrow-band imaging (ME-NBI), and endoscopic ultrasonography (EUS); however, each approach has its limitations [2]. Until now, there has been no consensus regarding the need for preoperative EUS. Several studies have suggested that the infiltration depth of EGC can be initially determined by WLE, and EUS is recommended only when it is difficult to determine the infiltration depth of gastric cancer by WLE [4–6]. Several studies have reported the accuracy of EUS in assessing the infiltration depth of EGC, with results ranging from 41.4–86% [7–10]. Especially in the upper third of the stomach, combined with ulcers or low-differentiated carcinoma, the diagnostic accuracy of EUS is low, which might easily lead to misdiagnosis [8].

According to the endoscopic diagnosis and treatment guideline, when a lesion is resected en bloc; is < 3 cm in diameter, predominantly differentiated type, pT1b (SM1, cancerous tissue confined to < 500 μm from the muscularis mucosae); has no lymphovascular infiltration; has negative surgical margins, curative resection is considered for expanded indications [11]. Surgical treatment is recommended for lesions with infiltration deeper than SM1. Thus, over-staging, in particular, tends to expose patients to unnecessary surgical trauma. Many studies have shown that the level of elevated mucosal lesions is related to the depth of infiltration [12]. The coexistence of EGC and benign submucosal lesions can imitate the illusion of submucosal infiltration, interfering with endoscopists’ judgement of the infiltration depth. Recently, our clinical work has found that collision EGCs have become more frequent; therefore, we present the first study to identify the characteristics of coexisting early gastric cancer and benign submucosal lesions.

## Methods

### Study design and population

This retrospective, multicenter, observational study was conducted at three hospitals (Qilu Hospital, Shandong University; Shandong Second Provincial General Hospital; and The First Affiliated Hospital of Shandong First Medical University). This study was approved by the Ethics Committee of Qilu Hospital, Shandong University, and was performed in accordance with the declaration of Helsinki (NO.2022-029).

We screened of patients suspected of EGC submucosal infiltration by WLE and ultimately selected for endoscopic submucosal dissection treatment after EUS and ME-NBI examination. Patients with early cancer infiltration above SM1 (confined to < 500 μm from the muscularis mucosae) and coexisting benign submucosal lesions were selected by tracking the pathological findings. Patients who selected surgical treatment, those with infiltration depth of EGCs exceeding SM2, and those with simple EGC confined to SM1 were excluded.

We retrospectively reviewed the medical records of all cases. Data such as age, sex, endoscopic performance, site and type of lesion, endoscopic ultrasonography results, and pathological findings were recorded.

We recruited eight endoscopists from three centers and divided them into two groups: experienced and inexperienced, according to the time and cases of endoscopic operations. The four experienced endoscopists (hereafter referred to, in no particular order, as A, B, C, and D, respectively) who had each performed endoscopy for at least 5 years, including > 3,000 endoscopic procedures each, and the other four endoscopists (E, F, G, and H, respectively) who had 1–3 years of endoscopic experience were considered inexperienced. Endoscopic white-light and narrow-band imaging magnified images of cases in this study (coexistence of EGC and benign submucosal lesions, EGC-SML group) and some EGCs (EGC group) were made into a test questionnaire for these eight physicians, who had not seen these cases before, to judge the depth of infiltration. The questionnaire consisted of a total of 45 cases and in addition to the 15 cases included in our study, there were 15 cases of EGC with infiltrative depth limited to within SM1 and 15 with infiltrative depth exceeding the SM1. Following a randomization of the order, eight physicians were asked to assess the infiltration depths and the results were analyzed.

### Statistical analysis

All statistical analyses were performed using GraphPad Prism, version 9.0.0 (GraphPad Software). The accuracy of the judgement of the infiltration depth was calculated, and the two groups were compared using the paired *t*-test. Statistical significance was set at *P* < 0.05.

## Results

**Fig. 1:**
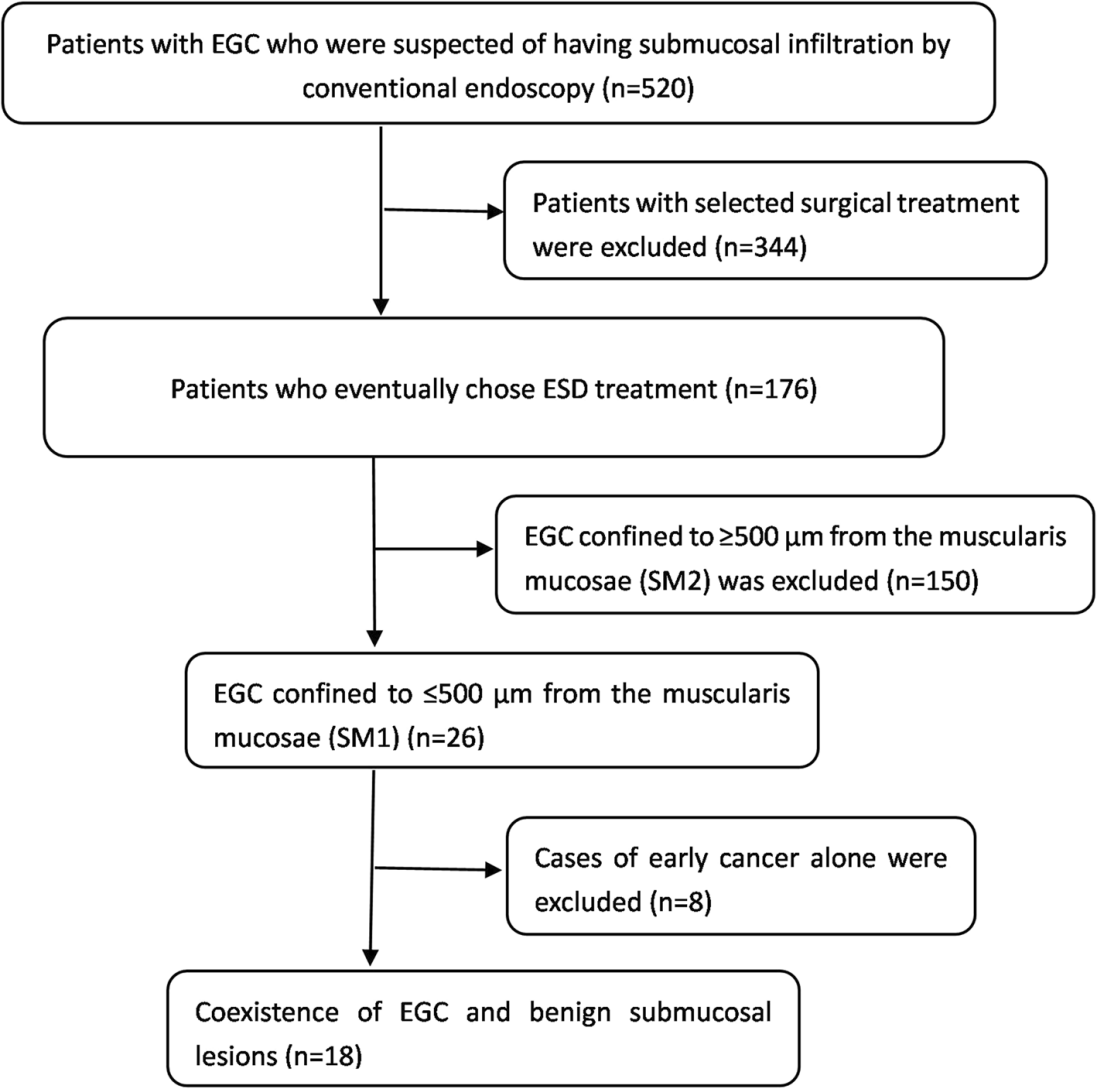
Flow diagram of the patients included in the study. EGC, early gastric cancer; ESD, endoscopic submucosal dissection

We screened 520 patients and retrospectively identified 18 EGC patients with invasive cancer-like morphology (Fig. 1); the characteristic information is presented in Table 1. We performed simple statistical analysis of the data (Tables 2 and 3). The patients consisted of 14 men and 4 women (male: female ratio = 3.5:1) with a mean age of 67.22 ± 7.14 years (range, 54–81 years). All patients were treated with en bloc ESD.

**Table 1:** Eighteen cases of coexistence of early gastric cancer and benign submucosal lesions

Case number	Age (years)/Sex	Lesion site	Type of mucosal lesion	Paris classification	Infiltration depth	Type of submucosal lesion	Performed EUS
1	67/F	Cardia	High-grade intraepithelial neoplasia	0-IIa	EP	Leiomyoma	Yes
2	63/F	Cardia	Intermediate-differentiated intramucosal adenocarcinoma	0-IIa	EP	Hamartoma-like hyperplasia	No
3	64/M	Cardia	High-grade intraepithelial neoplasia	0-IIa	EP	Pyloric gland ectopic	Yes
4	54/M	Cardia	High-grade intraepithelial neoplasia	0-Is	EP	Leiomyoma	Yes
5	69/M	Cardia	Low-differentiation adenocarcinoma	0-IIa	MM	Gastritis cystica profunda	No
6	71/M	Cardia	High-grade intraepithelial neoplasia	0-IIa + IIc	SM1	Gastritis cystica profunda	No
7	68/M	Cardia	High-grade intraepithelial neoplasia	0-IIa + IIc	EP	Gastritis cystica profunda	No
8	81/M	Cardia	Gastric foveolar epithelium dysplasia	0-IIa	EP	Gastritis cystica profunda	Yes
9	62/M	Cardia	Gastral tubular adenocarcinoma	0-IIa + IIc	SM1	Gastritis cystica profunda	Yes
10	61/M	Cardia	Gastral tubular adenocarcinoma	0-IIa + IIc	MM	Leiomyoma	No
11	80/M	Cardia	High-grade intraepithelial neoplasia	0-IIa + IIc	EP	Gastritis cystica profunda	No
12	67/M	Cardia	High-grade intraepithelial neoplasia	0-IIa	MM	Gastritis cystica profunda	No
13	63/F	Gastric body	Low-grade intraepithelial neoplasia	0-IIa + IIc	EP	Ectopic pancreas	Yes
14	66/F	Gastric body	High-grade intraepithelial neoplasia	0-IIa	EP	Gastritis cystica profunda	No
15	61/M	Gastric body	High-grade intraepithelial neoplasia	0-IIa	EP	Gastritis cystica profunda	Yes
16	77/M	Gastric body	High-grade intraepithelial neoplasia	0-IIa + IIc	MM	Gastritis cystica profunda	Yes
17	74/M	Gastric body	Intramucosal tubular adenocarcinoma	0-IIa	MM	Gastritis cystica profunda	Yes
18	62/M	Gastric body	Intramucosal tubular adenocarcinoma	0-IIa + IIc	MM	Gastritis cystica profunda	Yes

*F *Female, *M *Male, *EP *Epithelial mucosa, *LPM *Lamina propria mucosa, *MM *Muscularis mucosa, *SM1 *Confined to < 500 μm from the muscularis mucosae, *EUS *Endoscopic ultrasonography

**Table 2:** Patient and lesion characteristics

		Frequency	Percentage
Sex	Male	14	77.78
	Female	4	22.22
Performed EUS	Yes	10	55.56
	No	8	44.44
Lesion position	Cardia	12	66.67
	Gastric body	6	33.33
Type of mucosal lesion	High-grade intraepithelial neoplasia	10	55.56
	Low-grade intraepithelial neoplasia	2	11.11
	Gastral adenocarcinoma	6	33.33
Infiltrating depth	EP	10	55.56
	MM	6	33.33
	SM1	2	11.11
Paris classification	0-IIa	9	50
	0-IIa + IIc	8	44.45
	0-Is	1	5.55
Type of submucosal lesion	Gastritis cystica profunda	12	66.67
	Leiomyoma	3	16.68
	Hamartoma-like hyperplasia	1	5.55
	Pyloric gland ectopic	1	5.55
	Ectopic pancreas	1	5.55

*EP *Epithelial mucosa, *LPM *Lamina propria mucosa, *MM *Muscularis mucosa, *SM1 *Confined to < 500 μm from the muscularis mucosae, *EUS *Endoscopic ultrasonography

**Table 3:** Characteristics of coexistence of EGC and benign submucosal cystic lesions

		Cystic lesions (*n* = 13)
Position	Cardia	8 (61.54%)
	Gastric body	5 (38.46%)
Morphology (Paris classification)	0-IIa	7 (53.85%)
	0-IIa + IIc	6 (46.15%)
	0-Is	0 (0)
EUS	Definitive diagnosis	4 (30.77%)
	Indefinite diagnosis	3 (23.08%)
	Not performed	6 (46.15%)
Type of submucosal lesion	Gastritis cystica profunda	12 (92.31%)
	Pyloric gland ectopic	1 (7.69%)

*EGC *Early gastric cancer, *EUS *Endoscopic ultrasonography

Ten of the 18 patients underwent EUS, and submucosal lesions were found in 6 cases (ratio = 60%). The main lesion sites in these cases were the cardia (12/18, 66.67%), followed by the gastric body (6/18, 33.33%). High-grade intraepithelial neoplasia was the most common histopathological diagnosis (10/18, 55.56%), followed by gastric adenocarcinoma (6/18, 33.33%). The predominant morphologies were 0-IIa (9/18, 50.00%) and 0-IIa + IIc (8/18, 44.45%), according to the Paris classification. Most of the early cancer and precancerous lesions were confined to the mucosal layer (16/18, 88.89%), with a small percentage invading the submucosa (2/18, 11.11%).

**Fig. 2:**
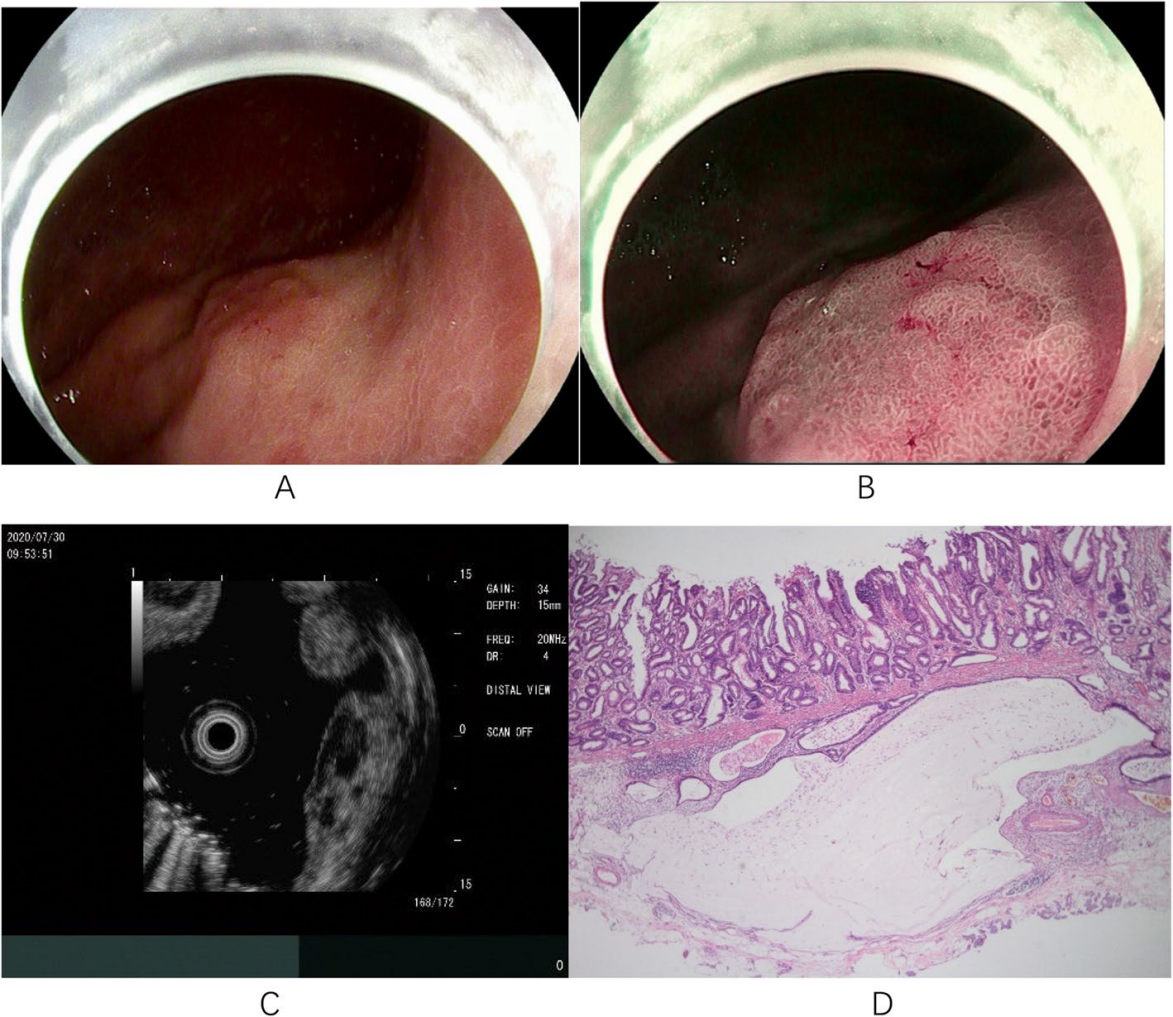
Case 15: **A** An elevated lesion measuring approximately 1.5x2.0 cm with a central depression, and a rough, red surface mucosa is seen on the posterior wall of the upper middle part of the gastric body. **B** Microglandular duct disorder and microvascular dilatation on magnification endoscopy. **C** The mucosal layer of the lesion is significantly thickened; the submucosal layer is slightly thickened; irregular hypoechoic clusters are visible within; and the intrinsic muscle layer is clear. **D** High-grade intraepithelial neoplasia of the mucosal layer combined with gastritis cystica profunda below (magnification 40x)

**Fig. 3:**
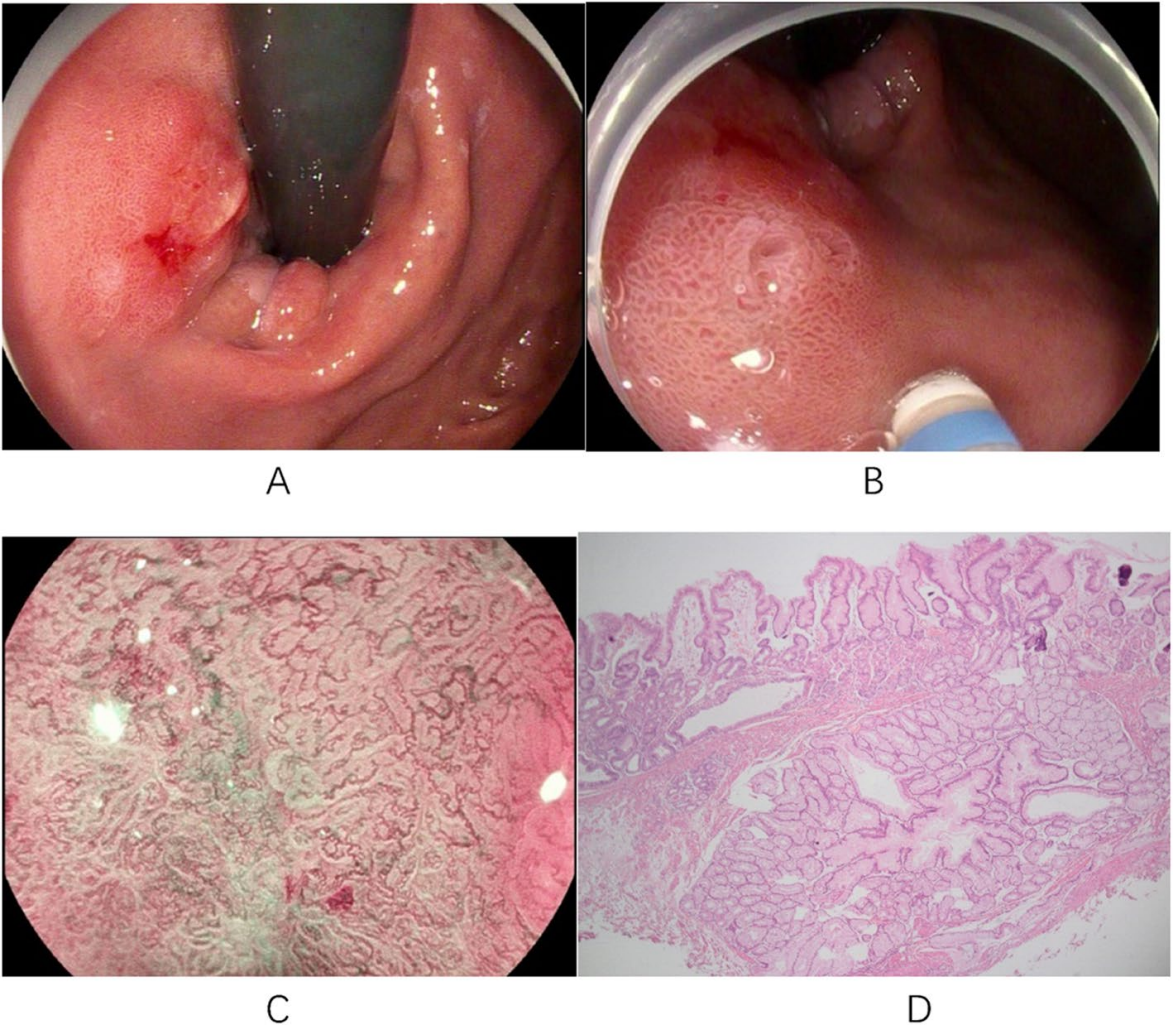
Case 3: **A** A 1.5x1.5cm type II-a lesion with mucosal hyperemia and erosion is seen on the less curved side of the cardia on white-light endoscopy. **B** The opening of the glandular duct can be seen at the edge of the lesion. **C** Microvascular and microglandular duct disorders seen on magnification endoscopy. **D** High-grade intraepithelial neoplasia of the mucosal layer and submucosal pyloric gland ectopic (magnification 40x)

**Fig. 4:**
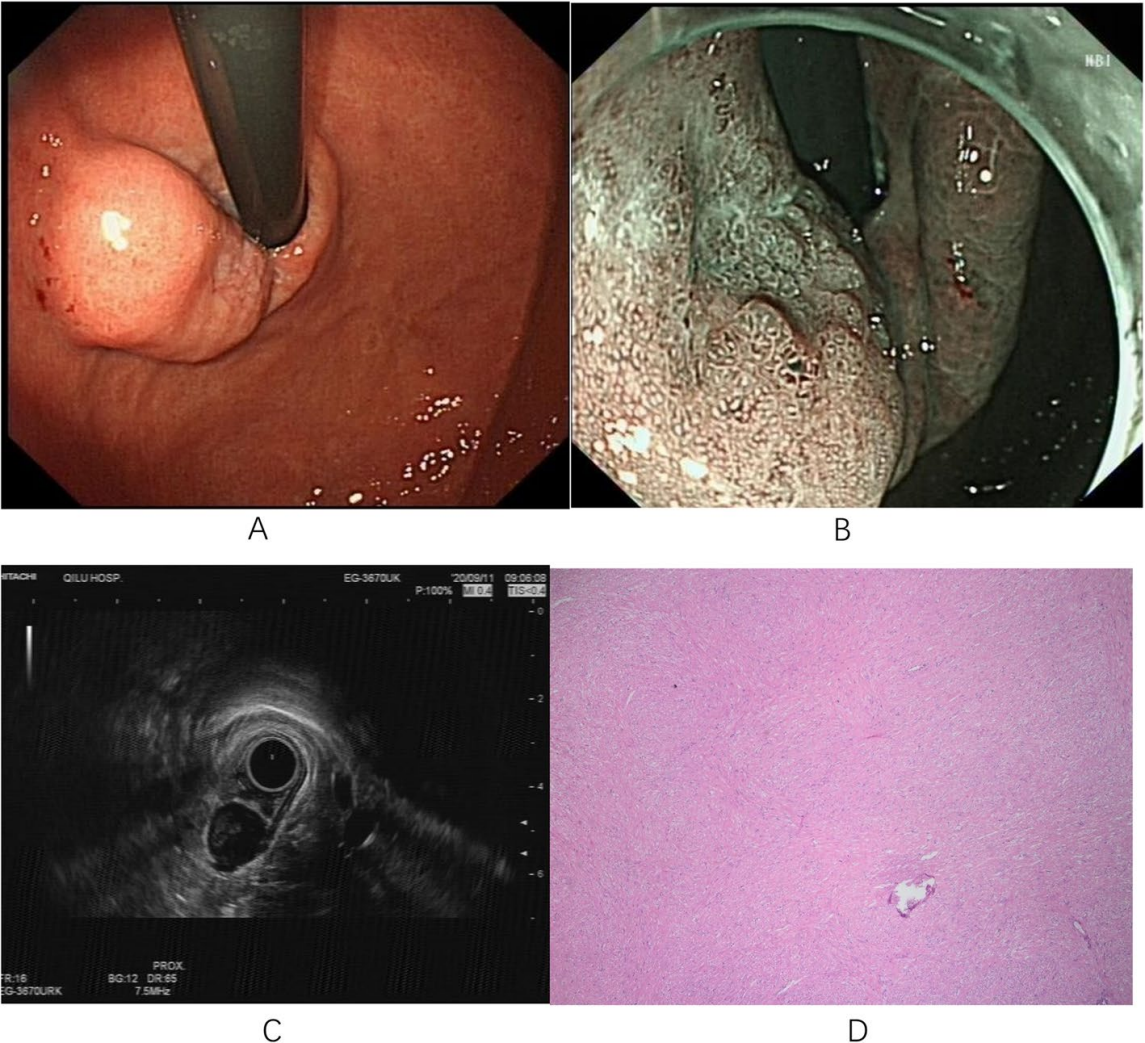
Case 4: **A** A 1.0x2.0cm elevated lesion on the posterior wall of the cardia with a rough mucosal surface and a slight central depression. **B** ME-NBI shows an increase in the microvascular diameter and irregular microglandular pattern. **C** Endoscopic ultrasonography scan showing a 2.0x1.6cm hypoechoic cluster with homogeneous internal echogenicity. **D** A leiomyoma in the submucosa (magnification 40x). ME-NBI, magnifying endoscopy with narrow-band imaging

**Fig. 5:**
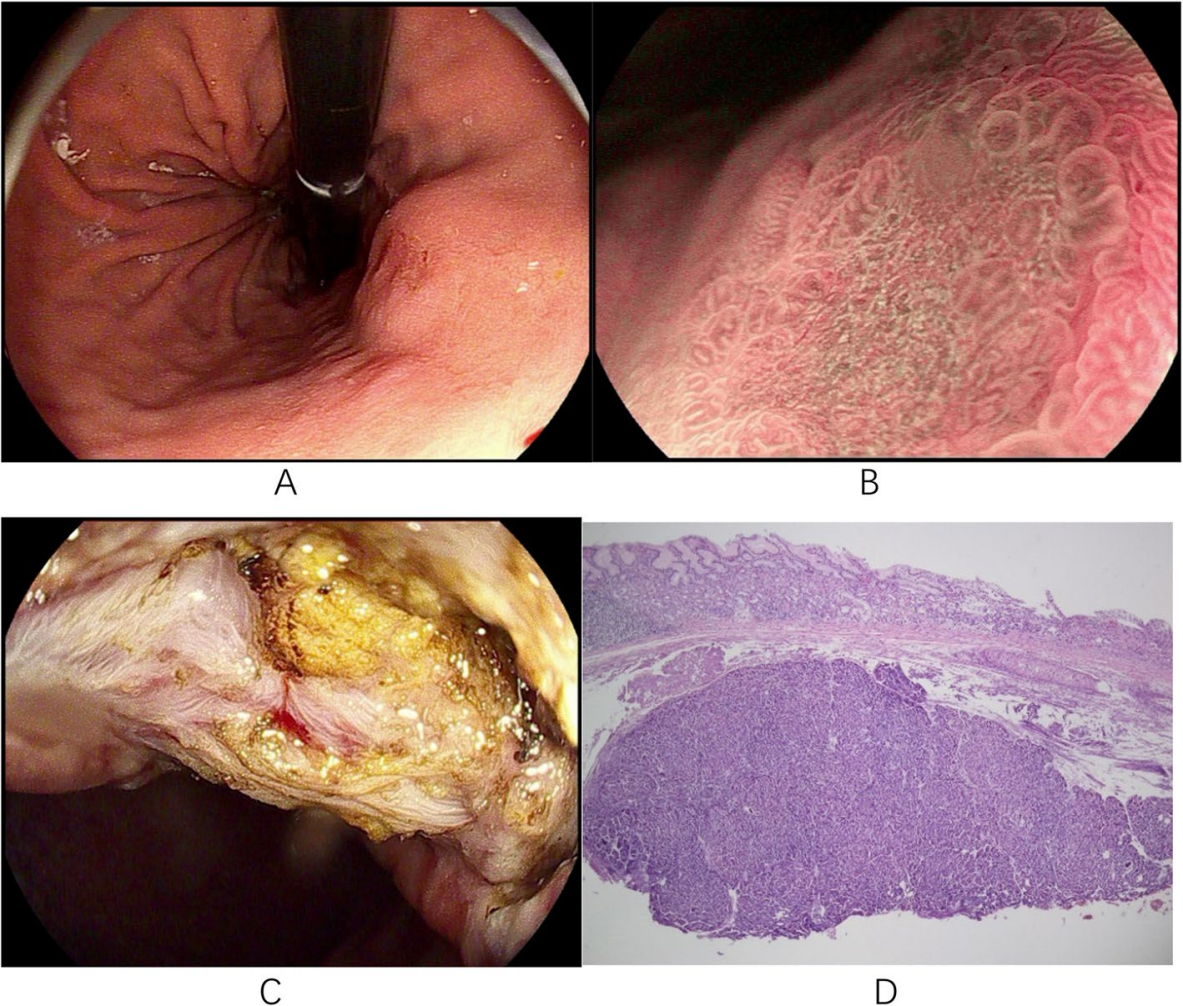
Case 13: **A** A type IIa+IIc lesion measuring approximately 2.0x3.0 cm is seen in the lower curvature of the gastric body with clear borders. **B** Magnification endoscopy showing a disorganized surface with a microvascular diameter and microglandular pattern. **C** A yellow tumor with indistinct borders is seen in the submucosa after dissection. **D** Low-grade intraepithelial neoplasia in the mucosal layer and submucosal ectopic pancreas (magnification 40x)

Based on different endoscopic and pathological features, coexisting submucosal lesions can be divided into two categories. One type was solid submucosal lesion (5/18, 27.78%), including leiomyoma (*n* = 3), hamartoma-like hyperplasia (*n* = 1), and ectopic pancreas (*n* = 1). This type of lesion is easier to diagnose by using conventional endoscopy and EUS. Another type of EGC combined with cystic submucosal lesions (13/18, 72.22%), such as gastritis cystica profunda (GCP) or ectopic pyloric glands, is more difficult to diagnose and can easily be confused with deep submucosal infiltration of EGCs. In these 13 cases, the cardia remained the most common site, and the Paris classification was type 0-II (0-IIa, 53.85%; 0-IIa + IIc, 46.15%); 12 cases (92.31%) of submucosal lesions were gastritis cystica profunda. Seven of these 13 patients underwent EUS, and only 4 cases were definitively diagnosed because of echoless structures in the submucosa. In the other 3 patients, the diagnosis was not confirmed because the EUS observation only revealed thickening of the submucosal layer without a distinct demarcation, which made it difficult to distinguish it from EGC submucosal infiltration. These three patients opted for diagnostic ESD, with definitive diagnosis relying on postoperative histopathological findings. We selected 4 typical cases for presentation (Figs. 2, 3, 4 and 5).

**Fig. 6:**
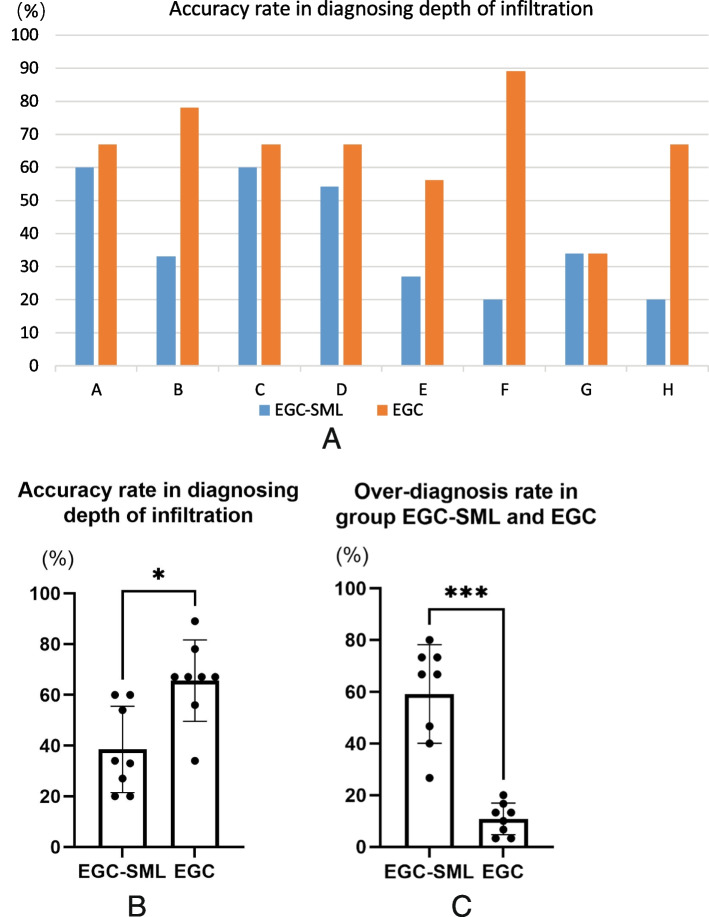
**A** Accuracy rate in diagnosing the EGC-SML and EGC infiltration depth for each endoscopist. **B** Comparison of the diagnostic accuracy between the EGC-SML and EGC group, *P*=0.0167. **C** Over-diagnosis rate in group EGC-SML and EGC, *P*<0.0001. EGC-SML, coexistence of early gastric cancer and benign submucosal lesions; EGC, simple early gastric cancer

Accuracy of the infiltration depth in the coexistence of EGC and benign submucosal lesions was significantly lower among experienced and inexperienced endoscopists than in EGC group (*P* = 0.0167, Fig. 6A, B). We analyzed two groups of misdiagnosis cases, in which the majority were over-diagnosed in the EGC-SML group, with a significantly higher proportion than in the EGC group (*P* < 0.0001, Fig. 6C).

## Discussion

This is the first study to summarize the clinical characteristics of coexistent EGC and benign submucosal lesions. Judgement of the infiltration depth in such cases is challenging for both experienced and inexperienced endoscopists. We found that these cases were important causes of inaccurate judgement and over-staging. In clinical practice, overestimation of the depth of EGC lesion infiltration leads to unnecessary surgery, whereas underestimation of the depth of infiltration increases the risk of secondary surgery.

For simple EGC, most studies and treatment guidelines recommend conventional endoscopy as the most effective method to determine the infiltration depth, whereas EUS should be used as an auxiliary method rather than a routine examination [13, 14]. From another perspective, EUS is the most effective method for the diagnosis of submucosal lesions. EUS can visualize submucosal lesions of the upper digestive tract and provide information regarding the layered structure of the digestive tract wall, originating layer of the lesions, and relationship between the lesion and surrounding tissues, peripheral lymph nodes, and adjacent organs [15, 16]. A retrospective study found that the diagnostic accuracies of EUS were 80.4% for stromal tumors and 68.0% for leiomyomas, with the highest diagnostic accuracy for lesions located in the muscularis mucosa [17]. However, the diagnosis of heterotopic pancreas, inflammation, benign cyst, glomus tumor, hamartoma, solitary fibroma, lymphangioma, angiogenic tumor, and angiolipoma using EUS is difficult because of their rarity and lack of characteristic features [17]. Accurate assessments of EUS are crucial for the diagnosis of these cases, and according to our study, the diagnostic accuracy of EUS for submucosal solid lesions is relatively high (100%), and all 3 cases of EGC coexisting with submucosal solid lesions were confirmed, which is consistent with previous reports. Submucosal cystic lesions and submucosal infiltration are indeed difficult to differentiate, and only 4 of the seven cases (57.14%) in which EUS was performed were properly diagnosed. Despite its lower diagnostic efficiency, EUS plays a valuable role in reducing the over-diagnosis of patients, and we recommend its use when suspecting such diseases.

In addition to common leiomyomas and lipomas, the types of submucosal lesions that coexist with early carcinomas include profound cystic gastritis, ectopic glands, and inverted polyps. In our study, there was a high percentage of GCP, especially lesions in the cardia area. GCP is common in elderly men and mainly located in the cardia and posterior and anterior walls of the gastric body; the results of our case are similar to those reported in the literature. Histopathological characteristics include gastric glands extending into the submucosal layer owing to hyperplasia and cystic dilatation [18]. GCP often presents as a submucosal tumor, solitary polyps, gastric mucosal fold, or even surface mucosa with no abnormal appearance [19, 20]. Although GCP is a benign lesion, approximately 3% of gastric cancers coexist with this lesion. This close association between GCP and malignancy suggests that GCP may be a pre-malignant lesion or a concurrent sharing of causative factors common to both disease conditions [20–22]. The diagnosis of EGC within GCP is difficult using endoscopy or biopsy. EUS is valuable for the endoscopic diagnosis of GCP, but it is often confused with other submucosal gastric lesions without typical manifestations [23]. It is primarily anechoic, mixed heterogeneous with thickened overlying mucosa, or hypoechoic with microcysts [19]. ESD is also an effective method for the diagnosis of such cases. Once GCP is detected, monitoring for EGC needs to be the focus.

**Fig. 7:**
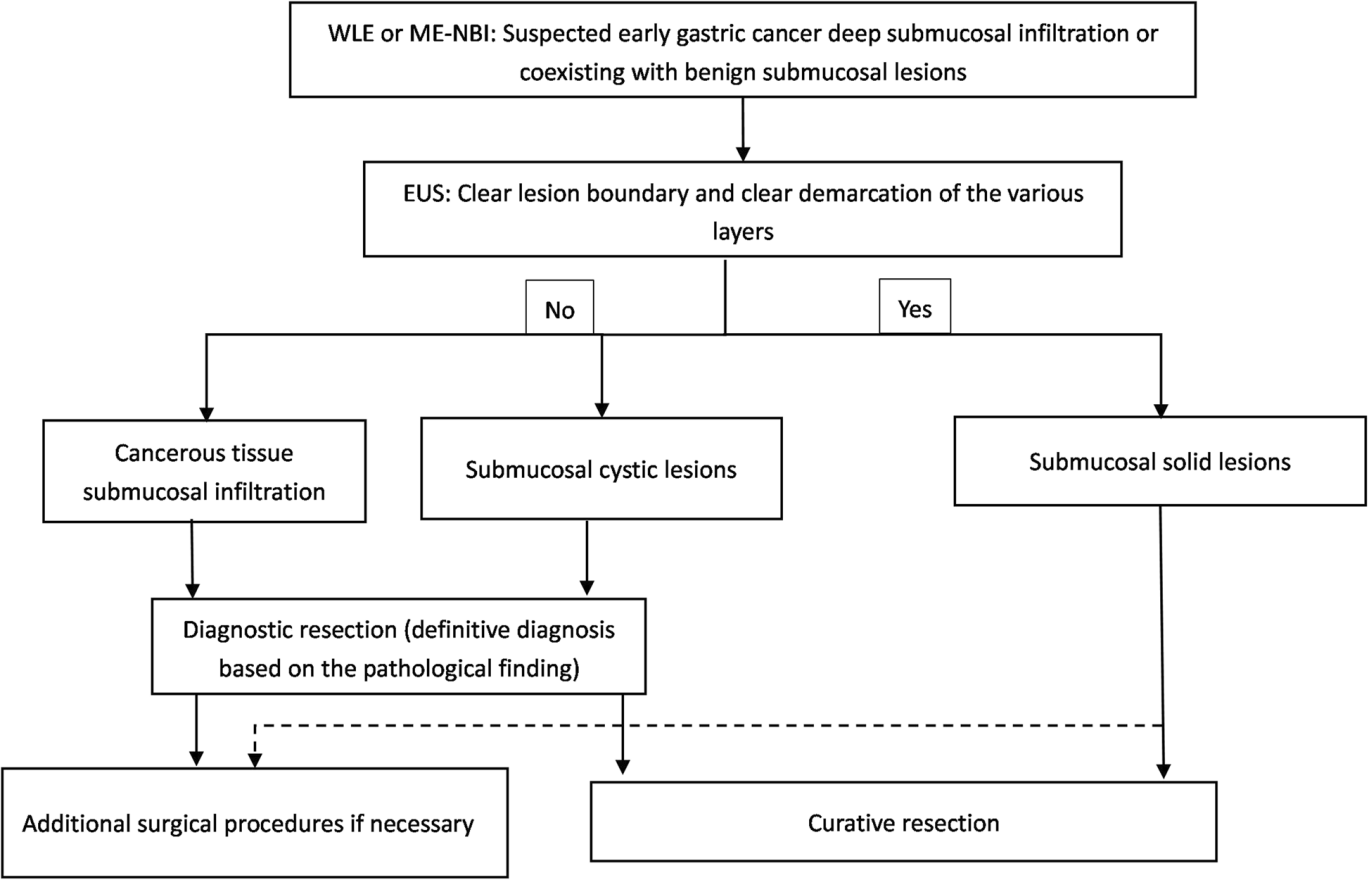
Flow diagram for the diagnosis of upper gastrointestinal early cancer with submucosal tumor-like morphology. WLE, white-light endoscopy; ME-NBI, magnifying endoscopy with narrow-band imaging; EUS, endoscopic ultrasonography

Since it is difficult to diagnose such diseases by endoscopy alone, all patients in this study underwent enhanced CT before ESD surgery, which provided suggestive information in only two cases of submucosal solid lesions, in other cases CT examination did not reveal valuable findings about submucosal lesions. This limitation can be attributed to factors such as the small size of the lesions, inadequate gastric filling in patients with low water intake, and the poor contrast of the contrast agent. CT and MRI do have significant limitations in predicting the depth of EGC infiltration. EGC with suspected deep infiltration requires careful selection of the treatment strategies. Based on our study, we designed a flow diagram for the diagnosis of coexisting EGC and benign submucosal lesions (Fig. 7). It seems more reasonable to consider that the two categories based on submucosal lesions are solid or cystic, with the former being easily diagnosed and the latter requiring careful differentiation. In cases where there is a suspicion of EGC coexisting with submucosal cystic lesions, the option of diagnostic endoscopic resection may be considered, with additional surgical procedures if necessary according to postoperative pathologic results. Based on the characteristics of the cases in this study, we have summarized five typical features that help to confirm the diagnosis when EGCs are combined with the following characteristics: (1) there are glandular duct openings on the surface of the lesion, and cystic fluid outflow is visible; (2) the lesion is located in the cardia; (3) the patient is an elderly male; (2) the boundary of the lesion is poorly defined on EUS, and an echogenic area is visible; and (4) the surface mucosa is mostly differentiated from early carcinomas or precancerous lesions. Among the 13 cases in this study, all patients (13/13,100%) satisfied two predictor factors, 10 (10/13, 76.9%) fulfilled three factors, and 3 (3/13, 30.8%) met four factors. The more characteristics that apply, the higher the possibility of suspected early cancer synchronous with submucosal cystic lesions; therefore, diagnostic ESD is recommended. In the future, we intend to augment the sample size to further validate.

This study has a couple of limitations. First, because the data were analyzed retrospectively, there may have been a selection bias. Second, a subgroup analysis between the submucosal solid and cystic groups could not be conducted because of the small number of patients. Our experiences mainly provide an suggestions for future directions, and further studies are needed to prove our inference.

## Conclusion

The coexistence of EGC and benign submucosal lesions is challenging for endoscopists because of the ease of overdiagnosis. Early cardiac-differentiated cancer with gastritis cystica profunda is the most common condition. In the diagnosis and treatment of EGC and precancerous lesions, no diagnostic criterion is absolute, and the more comprehensive the preoperative consideration, the more scientific the treatment plan.

## Data Availability

The datasets used and/or analysed during the current study available from the corresponding author on reasonable request.
